# The complex interplay of personal and external factors in medical students’ specialty decision-making: A qualitative study

**DOI:** 10.1371/journal.pone.0326932

**Published:** 2025-06-26

**Authors:** Quang Thanh Nguyen, Minh Phan Ngoc Nguyen, Nhi Yen Bui, Minh Quang Ngo, Linh Vu Thuy Nguyen, Trang Thu Dang

**Affiliations:** 1 College of Health Sciences, VinUniversity, Hanoi, Vietnam; 2 Department of Pediatric Surgery, Vietnam National Hospital of Pediatrics, Hanoi, Vietnam; Ho Chi Minh City University of Medicine and Pharmacy, VIETNAM

## Abstract

**Background:**

Medical students’ specialty decisions are shaped by a complex interaction of personal characteristics, academic experiences, social influences, and broader contextual factors. In lower-middle-income countries, where Western medical curricula are adapted to local educational and cultural contexts, little is known about how students make sense of these influences in shaping their future professional roles. This study aimed to explore how students interpret and construct their specialty choices in a newly established hybrid medical curriculum in Vietnam.

**Methods:**

This qualitative study used an interpretive phenomenological approach. Semi-structured interviews were conducted with 27 medical students across all four academic years at a Vietnamese medical school. Participants were selected through purposive sampling to ensure diverse academic backgrounds and training levels. Interviews were thematically analyzed using an inductive framework to identify how students experienced and interpreted influences on their specialty decisions.

**Results:**

Four interrelated themes emerged from the analysis. First, students described how personality traits shaped their emerging identity and influenced specialty preferences. Second, academic and extracurricular experiences, including clinical rotations, research, and volunteer work, were viewed as transformative encounters that clarified career direction. Third, students navigated financial hardship, family expectations, and academic pressure, often interpreting these constraints as factors in negotiating or compromising their career choices. Finally, the COVID-19 pandemic prompted moral reflection, with some students reaffirming frontline aspirations and others shifting toward specialties offering personal safety or opportunities for systemic impact.

**Conclusion:**

Medical specialty choice is a reflective and identity-driven process shaped by personal dispositions, experiential learning, social pressures, and contextual disruptions. Rather than being solely determined by traits or external incentives, students engage in an evolving interpretation of who they are and what kind of doctor they wish to become. Medical education should incorporate reflective support systems to guide students through this complex process.

## Background

Choosing a medical specialty is one of the most formative and emotionally charged decisions in a student’s professional life. It not only defines future scope of practice and lifestyle, but also reflects students’ evolving sense of self, purpose, and identity within the medical profession. While previous studies have examined specialty selection through frameworks of job security, income potential, or workforce alignment [1–5], these perspectives often overlook the lived complexity of how students interpret personal, academic, and social experiences in making these choices, particularly in culturally specific, resource-constrained contexts.

Specialty decision-making is not simply a cognitive task or response to external incentives; it is an interpretive process, unfolding as students encounter new experiences, form relationships with role models, and negotiate the tensions between idealism, institutional pressures, and family expectations. Intrinsic dispositions such as personality traits, engagement in academic and extracurricular activities, and the challenges of navigating medical training are not discrete variables, but deeply intertwined aspects of how students come to understand who they are and what kind of doctor they wish to become [6–8]. Yet, the literature remains limited in articulating how these elements function together, particularly from the students’ own perspectives in lower-middle-income countries (LMICs) [9,10].

In parallel, the COVID-19 pandemic has acted as an existential rupture in the professional lives of healthcare workers and trainees. Globally, medical students reported shifts in their perceptions of risk, vocation, and professional responsibility. For some, the crisis deepened their resolve to work in high-impact specialties; for others, it triggered withdrawal from frontline aspirations due to fear, moral distress, or burnout [11]. For example, Deng et al. [12] observed a reorientation of career interests toward fields perceived as more controllable or systemically vital, such as respiratory medicine. These evolving perspectives reflect not only external disruption but also internal processes of meaning-making, where career paths are reinterpreted in light of new realities.

Vietnam provides a particularly rich site for exploring these dynamics. As a lower-middle-income country undergoing rapid health system reform, it presents students with a juxtaposition of traditional hierarchies, familial expectations, and emerging global educational models. VinUniversity, established in collaboration with the University of Pennsylvania, delivers a blended, competency-based curriculum that merges Western pedagogical approaches with local cultural and institutional realities. In this hybrid environment, students engage with both global medical norms and culturally specific values, offering a unique opportunity to explore how professional identity and specialty preference evolve in an LMIC context.

Rather than reducing career decisions to fixed traits or linear causality, this study adopts an interpretive phenomenological approach introduced by Heidegger [13] and van Manen [14,15] to understand how medical students make sense of their journey toward a chosen specialty. We focus on the lived experiences that shape this process and examine how students interpret their personal dispositions, academic activities, social constraints, and contextual disruptions such as COVID-19 as part of a broader narrative of becoming a physician.

### Research objectives

This study aims to explore the following interpretive domains:

1. **Personality and Disposition**: To examine how students reflect on their personal characteristics and how these shape their emergent sense of professional identity.
2. **Engagement and Experience**: To understand the influence of academic and extracurricular involvement in shaping career inclinations and experiential learning.
3. **Constraint and Negotiation**: To explore the lived experience of financial, familial, and academic challenges, and how these pressures are interpreted in the context of specialty preference.
4. **Crisis and Reorientation**: To assess how the COVID-19 pandemic acted as a moral and existential turning point in students’ views of professional roles and future aspirations.

By investigating these dimensions, we aim to generate insights into the meaning structures underlying specialty decision-making and to inform faculty, advisors, and policymakers seeking to support students in developing careers that align with both their aspirations and their realities.

## Method

This study adopted an interpretive phenomenological approach, informed by the philosophical foundations of Martin Heidegger [13] and Max van Manen [14,15], to explore how medical students make sense of their emerging specialty preferences. Interpretive (hermeneutic) phenomenology is grounded in the understanding that individuals experience the world through the lens of personal meaning, social context, and situated being. Rather than bracketing assumptions, this approach emphasizes co-construction of meaning, recognizing the role of both participant and researcher in shaping interpretation. This design was appropriate for exploring the evolving sense of identity, value alignment, and moral reasoning that underpin career decision-making. This study followed the COREQ (Consolidated Criteria for Reporting Qualitative Research) guidelines to ensure methodological transparency, credibility, and depth.

### Research team

The research was conducted by a multidisciplinary team with expertise in medical education, qualitative research, and medical students’ career decision-making processes. The team was led by Dr. Nguyen (male) and Dr. Ha (female), faculty members at VinUniversity, holding MD and MSc degrees in Medical Education. Both have extensive experience in qualitative research and faculty development, ensuring the study’s methodological rigor. Five medical students, both male and female, trained in qualitative interview techniques, conducted the interviews under their supervision. The interviewers had no direct teaching responsibilities over the participants, minimizing power imbalances. Participants were informed that the researchers’ goal was to understand factors influencing specialty choices, motivated by Dr. Nguyen’s and Dr. Ha’s interests in improving medical education programs. Dr. Nguyen and Dr. Ha acknowledged their potential biases due to their backgrounds in pediatric surgery and medical education, respectively. They maintained reflexivity through field notes and analytic memos and engaged in peer debriefing to reduce bias and enhance credibility.

### Study design

We employed hermeneutic phenomenology as the methodological orientation to explore how students interpret and assign meaning to their dispositions, academic activities, external pressures, and pandemic experiences in shaping specialty preference. This approach was chosen to match the study’s aim of exploring identity development and meaning structures within medical training, rather than merely enumerating influencing factors.

### Participant selection

A purposive sampling strategy was used to recruit 27 medical students across all four cohorts (Years 1–4) of the Doctor of Medicine program at VinUniversity. The sample included students from preclinical (Years 1–3) and clinical (Year 4) phases to ensure a range of experiential depth and curricular exposure. Preclinical students, who had limited or no direct patient interaction, often relied on theoretical knowledge, early research participation, and student-led extracurricular activities to reflect on career interests. In contrast, clinical students had direct exposure to hospital-based practice and patient care, offering a more immersive and experiential basis for specialty preference. The participant pool included diverse perspectives in terms of gender, specialty interest, socioeconomic background, and degree of clinical exposure. This variety enriched the interpretive depth of the study, enabling us to examine how identity and meaning evolved across different stages of training. Participants were recruited through email invitations, online announcements, and learning platforms. Participation was voluntary, and all students provided informed consent. No participants withdrew or declined after enrollment.

### Data collection

Data collection occurred over two months (November 1, 2024, to December 30, 2024) to align with the academic calendar. Semi-structured, one-on-one interviews were conducted either in person or via secure video conferencing, based on participants’ availability and preferences. Each interview lasted 45–60 minutes and was audio-recorded with consent. Interviews were conducted in university rooms or participants’ homes. Field notes were taken to capture non-verbal cues, contextual elements, and immediate reflections. No third parties were present during the interviews, ensuring a private and secure environment. The initial interviews were sufficient for reaching data saturation, providing comprehensive insights without the need for repeat sessions. Participants were offered the opportunity to review their transcripts to validate the data captured and make any necessary corrections. Participant identifiers were assigned using an internal tracking system and are non-sequential to preserve confidentiality and facilitate secure data handling.

### Interview guide

The interview guide was developed through an extensive review of literature on specialty decision-making, identity development, and sociocultural influences in medical education. Thematic areas included:

1. Personal Dispositions: How do students describe their personality traits and moral values in relation to specialty fit?
2. Experiential Learning: What academic or extracurricular activities helped clarify or change their career inclinations?
3. Constraint and Negotiation: How do financial burdens, social expectations, and academic pressures influence their thinking?
4. Crisis Reorientation: How did the COVID-19 pandemic alter their views on frontline care and medical responsibility?

The guide underwent three layers of validation:

- Expert consultation: A panel of medical educators and qualitative researchers reviewed the guide to ensure alignment with interpretive aims.
- Pilot testing: Three students outside the final participant pool completed interviews to refine clarity, flow, and resonance of the questions.
- Iterative adjustment: As early themes emerged, the guide was adapted to probe deeper into moral conflict, identity formation, and contextual negotiation, hallmarks of phenomenological depth.

### Data analysis

Data were analyzed using a thematic approach informed by hermeneutic phenomenology and the six-phase model of Braun and Clarke. Rather than pre-coding for pre-existing constructs, the research team approached transcripts as texts of lived experience, searching for meaning units that reflected identity, transformation, or internal negotiation.

Two researchers independently coded transcripts using inductive, iterative cycles. Codes were grouped into interpretive themes that represented structures of lived meaning (e.g., “Becoming a certain kind of doctor”). These themes were refined through comparison, interpretive dialogue, and audit trails. Disagreements were resolved through discussion until consensus was reached. The software NVivo 12 supported coding organization and thematic mapping. Data saturation was considered reached when no new interpretive dimensions emerged from subsequent transcripts. Thematic structures were reviewed with selected participants and team members to confirm interpretive validity and enhance credibility.

### Ethical considerations

This study received ethical approval from the VinUniversity Institutional Review Board and Vinmec Healthcare System (No. 55/2024/QĐ-VMEC). Written informed consent was obtained from all participants prior to the interview. Data were anonymized, stored securely, and used exclusively for research purposes. Participant IDs were randomly assigned and do not reflect order or identity. Reflexivity and transparency were maintained throughout data handling and interpretation.

### Reporting and presentation of findings

Findings are presented using narrative excerpts that reflect the emotional and moral texture of students’ experiences. Each quote is attributed by participant ID, gender, and year of study. The Results section is structured around four existential themes: Becoming, Encounter, Negotiation, and Reorientation, that emerged as central to how students interpret their specialty choices.

The presentation emphasizes meaning-making over categorical reporting, demonstrating how career preferences are shaped not just by influences, but through students’ efforts to align who they are with who they wish to become in medicine.

## Result

### Participant demographics

The study included 27 medical students enrolled at VinUniversity across all four academic years of the Doctor of Medicine program. The sample comprised 15 female and 12 male students, ranging from Year 1 (preclinical phase) to Year 4 (clinical phase). Participants reflected a diverse range of socioeconomic backgrounds, career aspirations, and clinical exposure levels. Their intended specialties spanned across surgery, internal medicine, obstetrics & gynecology, psychiatry, pediatrics, dermatology, radiology, and family medicine, providing a wide spectrum of perspectives on specialty choice. This diversity enabled a rich, interpretive analysis of how personal and contextual factors influence the construction of professional identity throughout medical training.

Four interwoven, interpretively derived themes are presented: (1) identity alignment through personality traits, (2) transformation through academic and non-academic engagement, (3) the burden and negotiation of challenges, and (4) meaning-making in the wake of COVID-19. These themes illustrate not only what influenced students’ decisions, but how they interpreted, reconciled, and internalized these experiences in shaping their evolving professional identity.

1 **Identity alignment through personality traits**

Students reflected deeply on how intrinsic personality traits influenced their evolving specialty choices, not as isolated predictors, but as lenses through which they experienced medical training. Many described a sense of resonance between their personality and particular specialties, illustrating how specialty choice became a way of expressing identity.

For instance, students high in openness were drawn to dynamic, discovery-driven fields. Student ID-03 (Year 1, Male) shared, “Creativity and a desire to explore, rather than being overly outgoing, define me. I see myself in psychiatry where these traits are valuable”.

Similarly, Student ID-12 (Year 2, Female) expressed, “The knowledge in medicine is vast and endless... and I love learning new things,” describing how pediatrics reflected her insatiable curiosity.

Conscientiousness shaped decisions toward structured and disciplined fields. Student ID-15 (Year 3, Male) associated surgery with his drive for precision: “Good planning and self-discipline help me handle difficult situations better.” Student ID-21 (Year 4, Female) similarly felt that “internal medicine requires a methodical approach,” aligning with her conscientious nature.

Extraversion and agreeableness led students toward relational specialties. Student ID-07 (Year 1, Female) stated, “I enjoy spending time with people... which guides me toward family medicine,” while ID-25 (Year 4, Female) described geriatrics as a space for “gentle and understanding care.” Student ID-30 (Year 2, Male) shared, “I care deeply when someone is in a miserable condition... which draws me toward oncology.”

Neuroticism evoked tension between anxiety and precision. Student ID-18 (Year 3, Female) acknowledged, “I tend to worry, but that helps me care more,” finding dermatology’s predictability comforting. Likewise, ID-22 (Year 1, Female) noted, “Neuroticism allows me to focus on details,” making pathology an appropriate fit.

Through these reflections, students framed specialty choice not as personality-driven, but as identity-affirming—a process of seeking congruence between self and role.

2 **Transformation through academic and non-academic engagement**

Students described their academic and non-academic experiences as catalysts for reflection and identity clarification. These were not merely resume-building activities, but formative encounters that shaped how students interpreted their roles within medicine. In alignment with van Manen’s phenomenology of practice, such encounters can be viewed as pedagogical moments or opportunities through which learners articulate who they are becoming through what they do.

Some participants shared that a lack of direct medical engagement initially distanced them from clinical clarity but still provided unexpected value. Student ID-05 (Year 2, Male), who engaged in photography and debate clubs, reflected that while these activities didn’t guide his specialty decision directly, “they improved my observational skills and ability to engage in detailed discussions,” which he found relevant to dermatological assessment and communication. His reflection illustrates how non-clinical engagements may still support the interpretive lens through which students view medical work.

Clinical exposure emerged as a transformative experience that offered not just insight into the content of different specialties, but a deeper emotional and behavioral self-assessment. Student ID-22 (Year 1, Female), drawn to emergency medicine, explained, “Going to the hospital helped me understand each specialty more clearly,” emphasizing that these early exposures were clarifying rather than confirmatory. Similarly, ID-16 (Year 4, Male), who pursued surgery, shared, “My internships in surgical units were invaluable for skill development and career decisions,” suggesting that embodied practice reinforced both technical competency and vocational alignment.

Research involvement helped some students position themselves in intellectually intensive and inquiry-driven specialties. Student ID-14 (Year 3, Female), involved in oncology research, shared: “Research helps me understand clinical techniques better and broadens my relationships and experience.” For her, oncology was not just a technical interest, but a space where scientific inquiry and human engagement coexisted, offering a fulfilling career narrative.

Volunteerism and leadership in clubs contributed meaningfully to career imagination. Student ID-27 (Year 1, Male), active in community service, noted: “Joining clubs taught me management and communication skills, which shape my personality and future specialty choice.” His decision to pursue family medicine emerged from seeing himself as an educator and advocate in community health, a theme often overlooked in formal advising models.

Even academic competitions and enrichment tools proved influential. Student ID-09 (Year 2, Female), successful in neuroscience competitions, remarked: “Competitions helped me learn practical applications of neuroscience and research,” solidifying her commitment to neurology. Here, the act of mastering content through high-stakes engagement helped shape her future aspirations.

Taken together, these reflections show that students’ experiences with learning, leadership, and clinical engagement functioned not just as preparation for a specialty, but as sites of meaning-making, where they came to understand their values, strengths, and evolving professional selves.

3 **The burden and negotiation of challenges**

Students’ narratives revealed that challenges such as financial constraints, family expectations, academic intensity, and well-being, were not merely obstacles, but arenas of ethical and emotional negotiation.

Financial pressures were potent. Student ID-24 (Year 2, Male) said, “My family has to save a lot every month,” prompting a move toward surgery for financial return. In contrast, ID-17 (Year 3, Female), working part-time, sought a more balanced path in family medicine.

Family expectations created moral dissonance. ID-09 (Year 2, Female) disclosed, “My family expects me to pursue a prestigious specialty, even though I have different interests.” Though drawn to psychiatry, she contemplated surgery to fulfill those external hopes.

Academic overload shaped retreat from high-stress paths. ID-31 (Year 3, Male) admitted, “The academic workload is overwhelming... sometimes makes me want to quit,” pushing him toward dermatology. Well-being, too, was central. ID-26 (Year 2, Female) shared, “Long training time leaves me with little time for family and friends,” driving her toward specialties perceived to offer balance. These students interpreted their challenges not just as situational, but as shaping their professional integrity and future sustainability, often balancing vocation with vulnerability.

4 **Meaning-making in the wake of COVID-19**

The COVID-19 pandemic emerged as a profound and multifaceted influence in students’ reflections on specialty choice. It was not merely an external disruption, but a deeply felt experience that shaped students’ psychological states, motivations, values, and aspirations. Students described the pandemic as both a moment of existential uncertainty and a space of moral awakening, prompting reconsideration of frontline roles, redefinition of safety, and new engagement with systemic health issues.

Several students articulated a heightened sense of fear and psychological vulnerability during hospital placements, especially in high-contact environments. Student ID-34 (Year 3, Male) shared, “COVID-19 made me fearful every time I interned at the hospital,” conveying how exposure to clinical risk disrupted his sense of emotional security. For others, the fear was compounded by the risk to loved ones. ID-22 (Year 1, Female) expressed, “If I got infected, my family would face significant risks too,” while ID-27 (Year 1, Male) reflected, “Uncertainty during the pandemic made me rethink becoming a frontline doctor.” These experiences led many to reconsider or reject high-risk specialties such as emergency medicine, infectious diseases, or critical care.

Yet the pandemic also inspired students to reflect on the heroism, sacrifice, and societal importance of medical professionals. For some, this admiration translated into deeper vocational commitment. Student ID-18 (Year 3, Female) noted, “Seeing everyone come together to fight the pandemic made me admire doctors even more.” Student ID-15 (Year 3, Male) experienced a major career pivot: “Before COVID-19, I planned to study business. After working with nonprofits, I chose medicine,” highlighting how the pandemic influenced both prospective and enrolled students to reaffirm or initiate a medical calling.

A number of students also described a shift in focus toward lower-contact or more structured specialties, where they felt greater personal control and sustainability. Student ID-42 (Year 4, Male) stated, “The pandemic made me want to pursue medical research rather than frontline specialties,” while others turned to dermatology or pathology as fields offering lower exposure and higher predictability. Student ID-18 (Year 3, Female), previously drawn to frontline care, shifted her perspective: “I prioritize my family and work-life balance more now. I want a specialty where I can help others without sacrificing too much of myself.”

Some students moved beyond personal risk calculations to articulate a system-level critique of healthcare’s failures and inequities. Student ID-29 (Year 4, Female) said, “I want to change policies to protect healthcare workers better,” and ID-33 (Year 2, Male) reflected, “The lack of empathy in healthcare systems motivates me to improve patient-centered care.” These experiences reveal how the pandemic not only reconfigured individual specialty paths, but also instilled a commitment to systemic reform and advocacy.

Importantly, the decision to engage in or retreat from frontline care was often framed as a moral and existential consideration. Student ID-37 (Year 1, Female) expressed a strong ethical stance: “Being a doctor means thinking about others,” affirming her intention to pursue emergency medicine despite the risks. Conversely, students like ID-18 chose to prioritize family and self-preservation, revealing the valid tension between altruism and sustainability.

These narratives demonstrate how the pandemic acted as both a mirror and a magnifier, intensifying existing values while introducing new ethical tensions. Specialty choice during this period was deeply colored by emotional resonance, identity reflection, and moral clarity, transforming what might have been a practical decision into an existential one.

## Discussion

Medical students’ specialty choices result from a complex interplay of personality traits, academic and extracurricular experiences, external challenges, and the impact of the COVID-19 pandemic. These factors do not operate independently but interact dynamically, shaping students’ evolving career trajectories.

### Personality traits and specialty preferences

Personality traits emerged as interpretive anchors through which students made sense of their career inclinations and professional identity. Rather than simply determining specialty preference in a fixed or predictive manner, personality traits in this study were experienced and described as lived orientations to the world, shaping how students engaged with medical education, interpersonal relationships, and long-term professional goals. These findings respond directly to the first research objective: exploring how personal dispositions shape specialty preference and identity development.

Students who resonated with openness to experience described themselves as curious, imaginative, and eager to explore unknowns—qualities that led them to research-intensive and cognitively complex specialties such as psychiatry, oncology, and radiology. These students often articulated their vocational calling as a journey of discovery. One participant remarked: “The knowledge in medicine is vast and endless... and I love learning new things.” This aligns with prior findings by Kwon and Park [9], who reported that students high in openness are more likely to prefer medical fields requiring innovative thinking and deeper theoretical engagement.

Conscientiousness, marked by a preference for order, self-discipline, and reliability, was associated with specialties requiring structure, precision, and long-term commitment, especially surgery, internal medicine, and cardiology. In our study, conscientious students often juggled rigorous coursework with part-time employment, demonstrating resilience and goal orientation. These findings resonate with Judge et al. [16], who found conscientiousness to be a strong predictor of academic success and persistence in complex fields. However, Liu et al. [2] cautioned that while conscientiousness enhances knowledge acquisition, it may constrain the development of adaptive clinical skills due to over-structured learning behaviors. In our context, these students gravitated toward hands-on training environments, suggesting that pedagogical flexibility may help balance their strengths with real-world unpredictability. Turska et al. [17] also demonstrate that self-enhancement values, identified as the strongest positive predictor, along with conscientiousness, significantly influence medical students’ preference for surgical specialties.

Extraversion influenced students who valued communication, social energy, and high interaction roles. These students gravitated toward specialties such as pediatrics, emergency medicine, and family medicine, which are fields characterized by high interpersonal engagement. Several participants described clinical rotations and community service as pivotal moments where they realized their passion for patient-centered care. These findings echo Judge et al. [16] and Dehn and Eika [18], who emphasized the role of extraversion and social feedback loops in shaping medical career preferences. Notably, Dehn and Eika [18] also observed that such preferences are mediated by social and cultural contexts, suggesting that extraversion may be amplified or constrained in different institutional environments.

Agreeableness, associated with compassion, cooperation, and emotional attunement, was evident in students drawn to psychiatry, geriatrics, internal medicine, and family practice. These students expressed a desire to “care for the whole person” and to work in environments where empathy and continuity were central. This finding aligns with Santos et al. [19], who found that students with high empathy scores were more likely to choose people-oriented rather than technology-intensive specialties. Participants in our study described a strong emotional identification with vulnerable populations such as elderly, psychiatric, or chronically ill patients, suggesting that agreeableness operates not only as a trait but as a moral commitment in specialty preference.

Neuroticism, often associated with emotional sensitivity and stress reactivity, had a complex role in students’ reflections. While some students with higher neuroticism levels were deterred from high-risk or high-intensity specialties, others reinterpreted their anxiety as a strength—fueling meticulous preparation and diagnostic thoroughness. Students drawn to dermatology and pathology reported appreciating the predictability and lower emotional volatility of these specialties. These accounts mirror research by Hojat and Zuckerman [20], who found that lower neuroticism is more common in students selecting high-stress specialties such as surgery and emergency medicine, while higher neuroticism is associated with perceived susceptibility to health risks and preference for more controlled environments [21]. Our data suggests that students with higher neuroticism do not avoid clinical complexity, but rather seek contexts that support cognitive control, emotional regulation, and routine.

In sum, personality traits were not merely descriptors of student behavior but reflective tools through which students constructed and justified their specialty decisions. This interpretation aligns with van Manen’s view of professional development as a narrative process grounded in one’s being-in-the-world. These insights highlight the importance of integrating reflective personality frameworks into career counseling, ensuring that students do not feel constrained by trait categories but are encouraged to explore how their dispositions can evolve and adapt within various medical roles.

### Academic and extracurricular engagement as sites of meaning-making

Academic and extracurricular experiences emerged in this study as powerful interpretive moments or turning points through which students gained not only exposure to different specialties, but a deeper understanding of their own evolving identity, values, and career aspirations. These engagements, both formal and informal, provided context for self-reflection and shaped how students situated themselves within the broader medical profession. This theme addresses the second research objective: to understand how academic and extracurricular activities influence specialty preferences and professional development.

Clinical rotations and internships were widely described by students as formative experiences that confirmed or transformed initial interests. These immersive settings enabled students to interact with patients, observe team dynamics, and confront the realities of medical work in context. Many students reported that direct clinical exposure helped them “feel” their alignment, or misalignment, with specific fields. This supports findings by Ossai et al. [22], who reported that the majority of students made specialty decisions during clinical rotations. Similar results have been observed in broader literature, emphasizing the role of structured experiential learning in shaping medical students’ career clarity [12,22–24]. O’Doherty et al. [25] also shows that positive early clinical encounters, where students are given meaningful roles and included in patient care teams, promote a strong sense of belonging and professional competence. In this study, students high in openness particularly valued clinical exposure as a way to explore diverse fields before making commitments.

Research participation also proved pivotal, particularly for students inclined toward academic, diagnostic, and analytical specialties such as psychiatry, oncology, neurology, and pathology. Students described research as an opportunity to engage intellectually with complex topics and to explore medicine through a different lens, one grounded in inquiry and evidence. These findings are consistent with Souza et al. [3], who found that research was highly influential for students preferring neurology and psychiatry. Al-Johar et al. [26] further support this by demonstrating that students interested in neurology were motivated by the specialty’s analytical nature and the opportunity to engage in research, which they found intellectually stimulating. However, our results add nuance: research was not only a career influencer but a space of epistemological reflection, where students learned how they think and what kind of knowledge excites them. This is supported by Giske et al. [27], who found that students participating in clinical and research-like environments were challenged to critically examine how knowledge is constructed, evaluated, and applied—an experience that shaped both their understanding and emerging professional identities. Contrastingly, Wigney and Parker [28] noted that psychiatry is sometimes chosen for lifestyle reasons rather than academic passion; in our study, however, students expressed research-based interest and identity alignment with such specialties, suggesting a stronger motivational authenticity.

Students also described extracurricular activities, such as student organizations, volunteer work, and public health outreach, as essential to understanding their social roles as future physicians. These activities were described as sites of moral engagement, where values like empathy, leadership, and advocacy were tested and refined. For example, students active in community health programs felt affirmed in pursuing family medicine or primary care, citing emotional fulfillment and social connection. These findings echo those of Souza et al. [3], who observed correlations between specialty group participation and career interest in psychiatry and orthopedic surgery, and support theories that specialty choice is influenced by a dynamic interaction between personal values and perceived role characteristics [24,29].

In addition, students participating in academic competitions, particularly in neuroscience or anatomy, described these experiences as sharpening both interest and confidence. One student, who excelled in neuroscience competitions, expressed how these experiences solidified her interest in neurology through the excitement of applied problem-solving. These accounts suggest that competitive, problem-based learning can serve as a valuable tool for early identity exploration in medical education. Such engagements not only foster technical expertise but also allow students to test their cognitive style and professional inclinations in simulated real-world scenarios.

Taken together, these findings highlight that academic and extracurricular activities are not peripheral to medical training but are integral arenas of reflective practice, where students construct, test, and revise their understandings of what kind of doctor they wish to become. In line with interpretive phenomenology, these activities serve as phenomenological “spaces of becoming,” where knowledge, emotion, and professional identity converge.

### Navigating Financial, Social, and Academic Pressures

Students in this study described their specialty preferences not as static choices, but as evolving outcomes shaped by external demands, internal conflict, and adaptive self-reflection. Financial constraints, family expectations, and academic workload were not merely obstacles but moral and emotional terrains through which students interpreted their options and responsibilities. These findings speak directly to the third research aim: to examine how contextual challenges modify or reinforce students’ career decisions.

Financial difficulty was among the most recurrently cited influences in shaping career thinking. Several participants described juggling part-time work with academic responsibilities, leading them to consider specialties perceived as more financially rewarding or time-flexible. High-paying specialties such as surgery, anesthesiology, or dermatology were often chosen not from passion alone, but from a sense of familial duty, economic necessity, or debt management. This aligns with findings by Souza et al. [3], who identified financial security and controllable lifestyles as major factors influencing specialty choice. Phillips et al. [4] also found that students often considered high-paying specialties like surgery or anesthesiology not solely out of personal passion, but due to concerns about debt, future financial stability, and familial expectations. Moreover, in line with Kwon and Park [9], financial hardship remains a significant barrier not only to entering medicine but to navigating it with freedom of choice.

While some students, particularly those high in conscientiousness, found ways to sustain long-term specialty aspirations despite financial hardship, often through structured planning and resilience, others adopted a more risk-averse and self-preserving approach, favoring predictable specialties like dermatology or family medicine. These choices reflect an attempt to balance emotional regulation, future earning potential, and mental health. Students high in neuroticism were particularly sensitive to financial stress and preferred environments with less unpredictability and fewer acute demands, consistent with personality research linking neuroticism to stress avoidance [21].

Family expectations also shaped decision-making in complex and emotionally charged ways. In a sociocultural context such as Vietnam, where family prestige and communal values are deeply embedded, many students reported feeling torn between personal interest and familial obligation. This is further supported by Ha et al. [30], who found that, particularly in low- and middle-income countries (LMICs) in Asia, students often feel compelled to pursue prestigious specialties such as surgery or internal medicine due to familial pressure, even when their personal interests lie elsewhere. Specialties like psychiatry, geriatrics, or primary care were sometimes viewed by families as “less respectable,” while surgery, cardiology, and internal medicine were framed as markers of honor and success. One student shared that she struggled to justify her preference for psychiatry to her family, despite feeling emotionally drawn to the specialty. These narratives support the notion of symbolic capital described by Olsson et al. [31], where medical prestige is not merely individual achievement but a cultural currency that families invest in and expect returns from.

Such tensions often led to career compromises or internal conflict. Students recounted making peace with “pragmatic choices,” framing their final specialty decisions as moral negotiations between aspiration, duty, and dignity. For some, this tension reinforced existing choices; for others, it derailed a preferred path in favor of perceived familial or societal approval. These findings underscore the need for medical schools to create space for value-clarification exercises, cross-generational dialogue, and mentorship, especially for students experiencing divergence between personal desire and external expectation.

Academic workload and perceived institutional challenges further influenced specialty decisions. At VinUniversity, a newly established institution, some students expressed anxiety about the quality of training and the recognition of their degree in competitive specialties. As a result, they leaned toward specialties with shorter training pathways, lower competition, or more clearly defined postgraduate options. Several students explicitly described burnout and academic fatigue as reasons for selecting specialties with perceived lower intellectual content or emotional burden. This is consistent with Ferguson et al. [32], who identified the academic rigor and content of specialties as key decision-making factors. Similarly, Enoch et al. [33] found that students experiencing higher levels of burnout were more likely to choose specialties with more controllable lifestyles, such as dermatology or radiology, over more demanding fields like surgery or internal medicine.

Importantly, these pressures were not simply described as limitations but as meaningful experiences of vulnerability, realism, and identity negotiation. Students did not merely react to these stressors; they interpreted them as part of their becoming and integrating constraints into a coherent sense of self and future role. From this perspective, career decision-making was not an act of choosing among options, but a reflexive response to lived realities that carried moral, financial, and existential weight.

### Reorientation through crisis: COVID-19 and career reflection

The COVID-19 pandemic functioned as a transformative turning point for many students in this study, reshaping how they perceived risk, professional identity, and the moral dimensions of medical practice. In alignment with the fourth research objective, students’ specialty preferences during the pandemic were shaped not merely by situational factors, but by reflective meaning-making grounded in personal values, emotional capacity, and emerging professional aspirations.

For students high in conscientiousness, the crisis strengthened a sense of moral duty and drew them toward high-impact specialties such as emergency medicine and infectious diseases. These individuals interpreted the pandemic not as a deterrent, but as a call to service, where their future careers would contribute to broader societal resilience. This finding echoes those of Doan et al. [34], who observed increased student interest in frontline specialties like respiratory medicine during the pandemic, though with considerable variation in individual responses. A similar perspective is reflected in a descriptive phenomenological study by Mortazavi and Ghardashi [35], which found that morally driven students viewed the pandemic as an opportunity to serve society, reinforcing their commitment to socially impactful specialties.

Conversely, students with elevated neuroticism or caregiving responsibilities re-evaluated their risk tolerance, opting for specialties with lower patient contact and more controlled environments such as dermatology, radiology, or pathology. Rather than perceiving this shift as avoidance, these students framed their choices as efforts to maintain personal well-being and protect family members, highlighting the need for sustainable career pathways that accommodate both emotional and practical realities.

Some students, inspired by the heroism of healthcare workers, described a renewed motivation to pursue medicine, even if they had previously considered other career paths. The pandemic intensified their admiration for the profession and instilled a sense of purpose. In contrast, others responded by directing their interests toward systemic change, criticizing the structural gaps exposed by the crisis. These students expressed a desire to contribute through public health, health policy, or administrative leadership positions where they believed they could improve healthcare systems and support frontline workers.

These diverse responses align with findings from Doan et al. [34], who reported both increased motivation and reduced intent to remain in healthcare among students, depending on emotional reactions, social stigma, and perceived community support. This is further supported by Mortazavi and Ghardashi [35], whose phenomenological study showed that students’ emotional responses and caregiving roles led to divergent career tendency. Our study extends this understanding by revealing the interpretive depth behind these decisions where students were not only reacting to the crisis, but also reconstructing their professional identities in response to it.

Ultimately, the COVID-19 pandemic served as a moral lens, prompting students to reassess what it means to be a doctor, what kinds of risks they are willing to take, and how their personal values align with their professional futures.

### Implications for medical education and career counseling

The findings of this study highlight the complex, evolving nature of specialty decision-making among medical students, shaped not only by personality traits, activities, and contextual pressures, but by how students interpret and make sense of these influences in relation to their developing professional identity. Grounded in an interpretive phenomenological approach, our analysis revealed that students do not simply choose specialties, they construct them through meaning, reflection, and situated negotiation. Within this context, Bandura’s Social Cognitive Theory (SCT) [36] offers a valuable complementary lens for guiding educational interventions, particularly by addressing how environment, self-efficacy, and observed experience influence behavior and decision-making.

1 **Foster Reflective Career Counseling Rooted in Identity Formation**

Rather than relying on personality as a static predictor of specialty fit, advisors should engage students in reflective dialogues about how traits such as openness, conscientiousness, or agreeableness are experienced and expressed in clinical settings. Our findings showed that students interpreted their personality traits through the lens of lived experience—e.g., extroverts feeling affirmed in dynamic team settings, or conscientious students gravitating toward structured, outcome-driven specialties. Supporting students in reflecting on their traits-in-practice can enhance vocational clarity and help them articulate who they wish to become, not just what they are good at. SCT’s emphasis on self-efficacy further reinforces the importance of helping students recognize their personal capacity to succeed in roles that resonate with their values.

1. **Leverage Experiential Learning as a Site for Meaning-Making, Not Just Exposure**

Students in this study described clinical rotations, research, competitions, and community engagement as transformative spaces for identity construction. These experiences were more than resume-builders since they were moments of existential discovery. Medical schools should design curricula that not only offer varied exposure to specialties, but also incorporate structured reflection, narrative writing, and mentorship debriefings to help students process how these experiences inform their self-concept. As SCT suggests, such environments support observational learning and increase self-efficacy by allowing students to witness role models and internalize possible selves. From a phenomenological perspective, these moments are central to how professional identity is formed and reformed.

1. **Address Structural Pressures with Reflexivity and Support**

Financial hardship, family expectations, and academic fatigue emerged in this study as meaningful influences on career direction, often prompting students to reassess previously held ideals. Rather than viewing these factors as barriers to be removed, educators should acknowledge them as lived realities that shape moral and emotional choices. Counseling services must create space for students to explore the tension between external obligations and intrinsic motivation, including how cultural values, such as filial duty or prestige, impact career thinking. In line with SCT, this requires modifying the environment through scholarships, flexible program structures, and inclusive advising. In keeping with phenomenology, it also demands attentive listening and interpretive engagement with students’ life worlds.

4 **Integrate Crisis Reflection and Ethical Deliberation into the Curriculum**

The COVID-19 pandemic significantly influenced students’ self-perceptions and specialty choices—some experienced renewed motivation, others withdrew from frontline aspirations, and many reconsidered their long-term roles in medicine. These reactions were not uniform but reflected deep internal reorientation. Faculty development and curriculum design should incorporate opportunities for students to engage in collective meaning-making around crisis, including small-group discussions, storytelling, and simulated ethical dilemmas. SCT reinforces the importance of preparing students to develop competence and confidence under uncertainty, while phenomenology reminds us that professional formation is deeply linked to how individuals assign meaning to traumatic or disorienting experiences.

### Limitations and future directions

This study provides valuable insights into the multifaceted factors influencing medical students’ specialty preferences; however, several limitations must be acknowledged. First, as the study was conducted at a single institution, its findings may not be generalizable to other medical schools, particularly those with different educational structures or cultural contexts.

Second, while the study captures a broad range of factors influencing career choices, its cross-sectional design does not account for how these influences evolve over time. Longitudinal studies tracking students from medical school through residency and into early career stages could provide deeper insights into how initial specialty inclinations are reinforced or reshaped by real-world training experiences, mentorship, and professional challenges.

Given the complexity of specialty decision-making, future research should also examine the impact of different educational models on career choices. Understanding how students from diverse socioeconomic and cultural backgrounds navigate specialty selection will be essential for developing inclusive and equitable career counseling frameworks.

Additionally, there is also a pressing need for future research to evaluate and design evidence-informed, identity-sensitive career counseling frameworks. These should be grounded in students’ lived narratives, integrating personality reflection, structured mentorship, flexible financial support, and cultural responsiveness. Interventions that treat specialty selection as a process of becoming, rather than matching or forecasting, may better support students as they reconcile personal aspirations with systemic realities.

Finally, global health events such as the COVID-19 pandemic have shown how crisis can serve as a turning point in career reorientation. Future work should explore how reflective educational interventions, such as narrative medicine, moral case discussions, and crisis debriefing, might support students in processing disorienting experiences and integrating them into professional identity development.

## Conclusion

This study examined how medical students at a newly established institution in a lower-middle-income country make sense of their emerging specialty preferences through the lens of interpretive phenomenology. The findings reveal that specialty decision-making is a deeply reflective and identity-driven process, influenced by the interplay of personality traits, academic and extracurricular experiences, contextual constraints such as finances and family expectations, and disruptive events like the COVID-19 pandemic.

Rather than viewing these factors as static determinants, students engaged with them as part of a broader process of becoming, aligning who they are with the kind of doctors they wish to be. Clinical exposure, research, and volunteer activities served not only as practical experiences but as transformative moments that reshaped students’ sense of purpose and capacity. At the same time, external pressures prompted ethical deliberation and emotional recalibration, while the pandemic catalyzed both renewed commitment and strategic redirection.

These insights underscore the need for medical education and career counseling to move beyond trait-based guidance toward a more holistic, reflective, and context-sensitive model of professional development. Supporting students in navigating this complex journey will require integrated advising structures that recognize the social, emotional, and moral dimensions of specialty choice, ultimately fostering more authentic and sustainable career pathways in medicine.

## Supporting information

S1 File Study Questionnaires. (DOCX)

S2 File COREQ Checklist. (DOCX)

## Abbreviations

SCT: Albert Bandura’s Social Cognitive Theory
OB-GYN: Obstetrics and Gynecology
LMIC: Lower-Middle-Income Country

## References

[1] Sarikhani Y, Ghahramani S, Bayati M, Lotfi F, Bastani P. A thematic network for factors affecting the choice of specialty education by medical students: a scoping study in low-and middle-income countries. BMC Med Educ. 2021;21(1):99. doi: 10.1186/s12909-021-02539-5 33568113 PMC7877062

[2] Liu M, Cai J, Chen H, Shi L. Association of Personality Traits with Life and Work of Medical Students: An Integrative Review. Int J Environ Res Public Health. 2022;19(19):12376. doi: 10.3390/ijerph191912376 36231679 PMC9566667

[3] Correia Lima de Souza L, Mendonça VRR, Garcia GBC, Brandão EC, Barral-Netto M. Medical Specialty Choice and Related Factors of Brazilian Medical Students and Recent Doctors. PLoS One. 2015;10(7):e0133585. doi: 10.1371/journal.pone.0133585 26208007 PMC4514603

[4] Phillips JP, Wilbanks DM, Rodriguez-Salinas DF, Doberneck DM. Specialty income and career decision making: a qualitative study of medical student perceptions. Med Educ. 2019;53(6):593–604. doi: 10.1111/medu.13820 30821014

[5] Creed PA, Searle J, Rogers ME. Medical specialty prestige and lifestyle preferences for medical students. Soc Sci Med. 2010;71(6):1084–8. doi: 10.1016/j.socscimed.2010.06.027 20674118

[6] Schrepel C, Amick AE, Bann M, Watsjold B, Ilgen JS, Jauregui J. Self, Physician, and Specialty: A Qualitative Exploration of Medical Students’ Specialty Identity Formation During Undergraduate Medical Training. Acad Med. 2024;99(11):1184-90. doi: 10.1097/acm.0000000000005818 39475300 10.1097/ACM.0000000000005818

[7] Taylor AWR, Anderson ES, Gay S. “It’s a gamble”: A phenomenological exploration of medical students’ learning experiences as newcomers to clinical communities of practice. Clin Teach. 2024;21(4):e13708. doi: 10.1111/tct.13708 38058032

[8] Querido S, De Rond M, Wigersma L, van den Broek S, Ten Cate O. The Significance of Experiencing Clinical Responsibilities for Specialty Career Choice. Med Sci Educ. 2019;30(1):163–71. doi: 10.1007/s40670-019-00832-z 34457655 PMC8368942

[9] Kwon OY, Park SY. Specialty choice preference of medical students according to personality traits by Five-Factor Model. Korean J Med Educ. 2016;28(1):95–102. doi: 10.3946/kjme.2016.14 26838573 PMC4926941

[10] Saigal P, Takemura Y, Nishiue T, Fetters MD. Factors considered by medical students when formulating their specialty preferences in Japan: findings from a qualitative study. BMC Med Educ. 2007;7:31. doi: 10.1186/1472-6920-7-31 17848194 PMC2072940

[11] Luong V, Burm S, Bogie BJM, Cowley L, Klasen JM, MacLeod A, et al. A phenomenological exploration of the impact of COVID-19 on the medical education community. Med Educ. 2022;56(8):815–22. doi: 10.1111/medu.14793 35253255 PMC9115140

[12] Deng J, Que J, Wu S, Zhang Y, Liu J, Chen S, et al. Effects of COVID-19 on career and specialty choices among Chinese medical students. Med Educ Online. 2021;26(1):1913785. doi: 10.1080/10872981.2021.1913785 33849405 PMC8057072

[13] Heidegger M. Being and Time. First edition ed. Great Britain: Blackwell. 1962.

[14] van Manen M. Phenomenon of Practice. Second ed. New York: Routledge. 2023.

[15] van Manen M. Researching lived experience: Human science for an action sensitive pedagogy. 2 ed. New York: Routledge. 2016.

[16] Judge TA, Higgins CA, Thoresen CJ, Barrick MR. The big five personality traits, general mental ability, and career success across the life span. Personnel Psychol. 1999;52(3):621–52. doi: 10.1111/j.1744-6570.1999.tb00174.x

[17] Turska D, Skrzypek M, Tychmanowicz A, Baran T. Concept of distinct surgical personality revisited. Personality traits and personal values as surgical specialty choice predictors. European J Medical Technol. 2016;1(10):54–9.

[18] Dehn P, Eika B. Who’s choosing whom? A sociological study of the specialty choices in a Danish context. Int J Medical Education. 2011;2:36–43. doi: 10.5116/ijme.4dad.c33f

[19] Santos MA, Grosseman S, Morelli TC, Giuliano ICB, Erdmann TR. Empathy differences by gender and specialty preference in medical students: a study in Brazil. Int J Med Educ. 2016;7:149–53. doi: 10.5116/ijme.572f.115f 27213505 PMC4885636

[20] Hojat M, Zuckerman M. Personality and specialty interest in medical students. Med Teach. 2008;30(4):400–6. doi: 10.1080/01421590802043835 18569662

[21] Vollrath M, Knoch D, Cassano L. Personality, risky health behaviour, and perceived susceptibility to health risks. Eur J Pers. 1999;13(1):39–50. doi: 10.1002/(sici)1099-0984(199901/02)13:1<39::aid-per328>3.0.co;2-j

[22] Ossai EN, Uwakwe KA, Anyanwagu UC, Ibiok NC, Azuogu BN, Ekeke N. Specialty preferences among final year medical students in medical schools of southeast Nigeria: need for career guidance. BMC Med Educ. 2016;16(1):259. doi: 10.1186/s12909-016-0781-3 27716155 PMC5050581

[23] Rasic G, Hess DT, Richman AP, Pernar LI. Seeing is Believing - A Qualitative Study Exploring What Motivates Medical Students to Pursue a Career In General Surgery. J Surg Educ. 2024;81(8):1050–6. doi: 10.1016/j.jsurg.2024.05.016 38906788

[24] Kaminski A, Falls G, Parikh PP. Clerkship Experiences During Medical School: Influence on Specialty Decision. Med Sci Educ. 2021;31(3):1109–14. doi: 10.1007/s40670-021-01281-3 34457954 PMC8368383

[25] O’Doherty D, Culhane A, O’Doherty J, Harney S, Glynn L, McKeague H, et al. Medical students and clinical placements - a qualitative study of the continuum of professional identity formation. Educ Prim Care. 2021;32(4):202–10. doi: 10.1080/14739879.2021.1879684 33583348

[26] Al-Johar ZA, Hatem AK, Abbas HM, Khaled HW, Othman MT. Motivation for Choosing Neurology as a Career, among Students of Baghdad Medical College. Medico Legal Update. 2020;20(4):1822-8. doi: 10.37506/mlu.v20i4.2107

[27] Giske S, Kvangarsnes M, Landstad BJ, Hole T, Dahl BM. Medical students’ learning experience and participation in communities of practice at municipal emergency care units in the primary health care system: a qualitative study. BMC Med Educ. 2022;22(1):427. doi: 10.1186/s12909-022-03492-7 35655298 PMC9164765

[28] Wigney T, Parker G. Factors encouraging medical students to a career in psychiatry: qualitative analysis. Aust N Z J Psychiatry. 2008;42(6):520–5. doi: 10.1080/00048670802050637 18465379

[29] Querido SJ, Vergouw D, Wigersma L, Batenburg RS, De Rond MEJ, Ten Cate OTJ. Dynamics of career choice among students in undergraduate medical courses. A BEME systematic review: BEME Guide No. 33. Med Teach. 2016;38(1):18–29. doi: 10.3109/0142159X.2015.1074990 26372112

[30] Ha MT, Dao HC, Nguyen THH, Nguyen HL, Le P. Examining dimensions of career intentions: insights from medical and nursing students at a private not-for profit university in Vietnam. BMC Med Educ. 2024;24(1):1303. doi: 10.1186/s12909-024-06028-3 39538220 PMC11562080

[31] Olsson C, Kalén S, Ponzer S. Sociological analysis of the medical field: using Bourdieu to understand the processes preceding medical doctors’ specialty choice and the influence of perceived status and other forms of symbolic capital on their choices. Adv Health Sci Educ Theory Pract. 2019;24(3):443–57. doi: 10.1007/s10459-018-09872-3 30656525 PMC6647503

[32] Ferguson E, Semper H, Yates J, Fitzgerald JE, Skatova A, James D. The “dark side” and “bright side” of personality: when too much conscientiousness and too little anxiety are detrimental with respect to the acquisition of medical knowledge and skill. PLoS One. 2014;9(2):e88606. doi: 10.1371/journal.pone.0088606 24586353 PMC3937323

[33] Enoch L, Chibnall JT, Schindler DL, Slavin SJ. Association of medical student burnout with residency specialty choice. Med Educ. 2013;47(2):173–81. doi: 10.1111/medu.12083 23323656

[34] Doan LP, Dam VAT, Boyer L, Auquier P, Fond G, Tran B, et al. Impacts of COVID-19 on career choices in health professionals and medical students. BMC Med Educ. 2023;23(1):387. doi: 10.1186/s12909-023-04328-8 37237404 PMC10215048

[35] Mortazavi F, Ghardashi F. Medical students’ psychological and behavioral responses to the COVID-19 pandemic: A descriptive phenomenological study. Clin Child Psychol Psychiatry. 2022;27(1):291–307. doi: 10.1177/13591045211056922 34865547 PMC8819557

[36] Bandura A. Social foundations of thought and action: A social cognitive theory. Englewood Cliffs, NJ, US: Prentice-Hall, Inc. 1986.

