# Supplementary material for: The complex interplay of personal and external factors in medical students’ specialty decision-making: A qualitative study
Source: PLoS One. 2025 Jun 26;20(6):e0326932. doi: 10.1371/journal.pone.0326932 (PMC12200644; doi:10.1371/journal.pone.0326932)
Supplement: S1 File — (DOCX) [file pone.0326932.s001.docx]

**INTERVIEW GUIDE WITH PROBING QUESTIONS**

**Introduction & Obtain Consent**

**Welcome & Purpose**

- **Welcome Statement**:
  *"Thank you very much for taking the time to speak with us today. Your insights and experiences are invaluable to our study and will greatly contribute to our understanding of the factors influencing medical specialty choices."*
- **Study Purpose**:
  *"Our study explores the various factors that influence medical students like yourself in choosing a specialty. We want to understand how personal preferences, academic experiences, and external pressures shape career decisions. We are also examining how recent events, such as the COVID-19 pandemic, have impacted medical students’ views on different specialties. The insights gained from this research will help medical educators and policymakers better support students in making well-informed career choices."*
- **Confidentiality Assurance**:
  *"We want to reassure you that all information provided will remain confidential and will be used solely for research purposes. If you have any further questions about the study or wish to receive a summary of our findings, please do not hesitate to contact us."*
- **Consent Confirmation**:
  *"Before we begin, do you have any questions or concerns?"*

**Demographic Questions**

1. **Year of Study**
   - *"What year of study are you currently in?"*
   - **Probing**:
     - *"How has your current stage of training influenced your thoughts about specialty choices?"*
2. **Specialty Preference**
   - *"Have you decided on a specialty? If yes, which one? If no, what are your top considerations?"*
   - **Probing**:
     - *"What aspects of this specialty appeal to you most?"*
     - *"Have your preferences changed since entering medical school?"*
3. **Background Information**
   - *"Can you tell me a bit about your background (e.g., previous education, interests)?"*

**Main Interview Topics**

**1. Influence of Personality Traits on Specialty Choice**

- *"Have you ever thought about how your personality influences your specialty preference?"*
- **Explanation of Personality Traits (if needed):**
  - **Openness** (*Curiosity, creativity, willingness to explore new ideas and experiences*)
  - **Conscientiousness** (*Discipline, organization, and precision in tasks and decision-making*)
  - **Extraversion** (*Sociability, enjoyment of teamwork, high energy in group settings*)
  - **Agreeableness** (*Empathy, compassion, and preference for patient-centered interactions*)
  - **Neuroticism** (*Emotional sensitivity, anxiety, preference for structured and controlled environments*)
- **Probing**:
  - *"Do you think any of these traits have influenced your decision? If so, how?"*
  - *"Can you give an example of a moment where your personality trait strongly influenced your career interest?"*
  - *"Do you feel your personality aligns better with certain specialties? Why or why not?"*

**2. Influence of Academic & Non-Academic Activities**

- *"Which academic and non-academic activities do you think matter most in your choice of specialty?"*
- **Probing (Academic Activities):**
  - *"What coursework or clinical experiences have influenced your decision?"*
  - *"Have any particular professors, rotations, or classes shaped your interest?"*
  - *"Did participating in research impact your specialty choice?"*
- **Probing (Non-Academic Activities):**
  - *"How have extracurricular activities (clubs, leadership roles, volunteer work) influenced your preferences?"*
  - *"Have competitions or mentorship programs played a role in shaping your career decision?"*

**3. Challenges Faced During Medical Training (Financial, Social, and Academic Pressures)**

- *"Can you describe any challenges that you or your family have faced that might impact your decision to pursue a medical career or a particular specialty?"*
- **Probing (Financial Challenges):**
  - *"How do financial considerations influence your choice of specialty?"*
  - *"Are there specialties you feel are more financially viable given your personal or family economic situation?"*
- **Probing (Family/Social Pressures):**
  - *"Have family expectations influenced your choice of specialty?"*
  - *"Do you feel pressure to pursue certain specialties due to prestige, financial rewards, or job security?"*
- **Probing (Academic Workload & Well-being):**
  - *"Have you found certain specialties too academically or emotionally demanding?"*
  - *"Has your medical school experience changed how you perceive different specialties?"*

**4. Impact of COVID-19 on Specialty Choice**

- *"How did the COVID-19 pandemic impact your choice of specialty?"*
- **Probing:**
  - *"Did it change your perception of frontline specialties like emergency medicine or infectious diseases?"*
  - *"Did you reconsider certain specialties due to concerns about work-life balance, mental health, or personal safety?"*
  - *"Did the pandemic highlight any healthcare system weaknesses that influenced your career path?"*
  - *"Did COVID-19 inspire you to pursue research, public health, or policy-making in medicine?"*

**5. Willingness to Work as a Frontline Doctor**

- *"Are you willing to be a frontline doctor if there is another pandemic?"*
- **Probing:**
  - *"On a scale of 1-5, how certain are you?"*
  - *"What factors influenced your response?"*
  - *"Did the pandemic shape your views on high-stress medical roles?"*

**6. Influence of Mentorship & Role Models**

- *"Have mentors or role models influenced your specialty decision?"*
- **Probing:**
  - *"What qualities or advice from mentors guided you?"*
  - *"Did you have the opportunity to shadow professionals in your preferred specialty?"*
  - *"Was there a particular moment with a mentor that shaped your career decision?"*

**7. Perception of Work-Life Balance in Specialty Choice**

- *"How important is work-life balance in your decision-making process?"*
- **Probing:**
  - *"Have you considered how your specialty choice might impact your personal and family life?"*
  - *"Are there specialties you avoided due to work-life balance concerns?"*

**8. Long-Term Career Goals**

- *"Where do you see yourself in 10 years? How does this vision influence your specialty preference?"*
- **Probing:**
  - *"Do you prioritize personal satisfaction, financial stability, or work-life balance?"*
  - *"How does your long-term vision align with the demands of your preferred specialty?"*

**Closing Questions & Reflection**

1. **Reflection on Specialty Decision**
   - *"Looking back, is there anything you wish you had done differently in preparing for your future specialty?"*
   - **Probing:**
     - *"Are there activities or experiences you would recommend to other students making this decision?"*
2. **General Feedback on the Interview**
   - *"Is there anything else you would like to share about your experiences or thoughts on this topic?"*
   - **Probing:**
     - *"Do you feel this interview captured your perspectives accurately?"*

**Closing Remarks**

*"If you have any more thoughts after today’s session, please feel free to reach out to us using the contact information provided at the start of this session. Your participation is greatly appreciated and contributes to improving medical education and career guidance for future students. Thank you again, and we wish you the best in your medical journey!"*
